# A new GFP-tagged line reveals unexpected Otx2 protein localization in retinal photoreceptors

**DOI:** 10.1186/1471-213X-7-122

**Published:** 2007-11-02

**Authors:** Nicolas Fossat, Coralie Le Greneur, Francis Béby, Stéphane Vincent, Pierre Godement, Gilles Chatelain, Thomas Lamonerie

**Affiliations:** 1 IGFL, UMR CNRS 5242-INRA 1237-ENS, IFR128 Lyon-Gerland, 46 allée d'Italie, 69364 Lyon Cedex 07, France; 2 LBMC, UMR CNRS 5239 -ENS, IFR128 Lyon-Gerland, 46 allée d'Italie, 69364 Lyon Cedex 07, France; 3 Institut Pasteur, CNRS URA 2578, 28 rue du Docteur Roux, 75724 Paris cedex 15, France; 4 Embryology Unit, Children's Medical Research Institute, University of Sydney, Westmead, NSW 2145, Australia

## Abstract

**Background:**

Dynamic monitoring of protein expression and localization is fundamental to the understanding of biological processes. The paired-class homeodomain-containing transcription factor Otx2 is essential for normal head and brain development in vertebrates. Recent conditional knockout studies have pointed to multiple roles of this protein during late development and post-natal life. Yet, later expression and functions remain poorly characterized as specific reagents to detect the protein at any stage of development are still missing.

**Results:**

We generated a new mouse line harbouring an insertion of the GFP gene within the Otx2 coding sequence to monitor the gene activity while preserving most of its functions. Our results demonstrate that this line represents a convenient tool to capture the dynamics of *Otx2 *gene expression from early embryonic stages to adulthood. In addition, we could visualize the intracellular location of Otx2 protein. In the retina, we reinterpret the former view of protein distribution and show a further level of regulation of intranuclear protein localization, which depends on the cell type.

**Conclusion:**

The GFP-tagged *Otx2 *mouse line fully recapitulates previously known expression patterns and brings additional accuracy and easiness of detection of *Otx2 *gene activity. This opens up the way to live imaging of a highly dynamic actor of brain development and can be adapted to any mutant background to probe for genetic interaction between *Otx2 *and the mutated gene.

## Background

Studying the expression and intracellular localization of transcription factors is a difficult task because both may be highly dynamic. This is precisely the case for Otx2. Mouse *Otx2 *is a *paired-class *homeobox gene that belongs to a gene family also containing *Otx1 *and the more divergent *Crx *[1]. It plays critical roles in early brain induction and development [2]. It is expressed in a very dynamic fashion in areas of the central nervous system (CNS) that rapidly change as development proceeds [3-5]. Several germinal and conditional knock-out studies have emphasized its involvement in multiple functions such as head formation [6-8], photoreceptor fate determination [9] or post-natal survival and growth [10].

Despite the great number of genetic models generated to address its activities, there is still a need for tools to study the complex dynamics of expression of this gene in the CNS. Indeed, one major problem is the lack of specific Otx2 antibody. The strong sequence similarity between Otx1 and Otx2 has made it difficult to raise specific sera, the use of which is therefore particularly delicate [4,11]. The distribution of Otx2 protein has been first investigated during mouse development [4] with a polyclonal serum that was later shown to cross react with Otx1 protein [12] and then in newborn rats [11]. In adults, aside from few studies such as in the retina [13], no general study has been carried out. As a result, unambiguous *Otx2 *expression data mostly rely on mRNA detection [5,14]. *Otx2 in situ *hybridization (ISH) is widely used to characterize the patterning and development of anterior neuroectoderm. However, several studies have raised the possibility of translational regulation at gastrula stage [15,16], and recent work suggested that *Otx2 *specific miRNA decay might time the generation of retinal neurons [17]. Therefore, true Otx2 expression analysis should rely on protein rather than mRNA detection. Several LacZ reporter lines have been created [6,18] but due to probable deletion of splicing and regulatory sequences [19] or mRNA nonsense mediated decay, none of them allowed the complete monitoring of *Otx2 *gene expression. In addition, these models do not allow the precise determination of Otx2 protein intracellular localization. Yet, this appears to be tightly regulated in the developing retina [13,20]. Moreover it would be interesting to be able to examine OTX2 expression and detailed localization, and to identify directly Otx2-expressing cells, in live tissues.

To overcome these difficulties, we have generated a new mouse line harbouring a GFP tag within *Otx2 *natural genome context. Genetic modifications were made to ensure as a normal expression as possible. This line allowed to visualize the full Otx2 development pattern and to discover an unexpected control of Otx2 protein subcellular localization.

## Results

### Generation of an Otx2^+/Otx2-GFP ^reporter line

**Figure 1:**
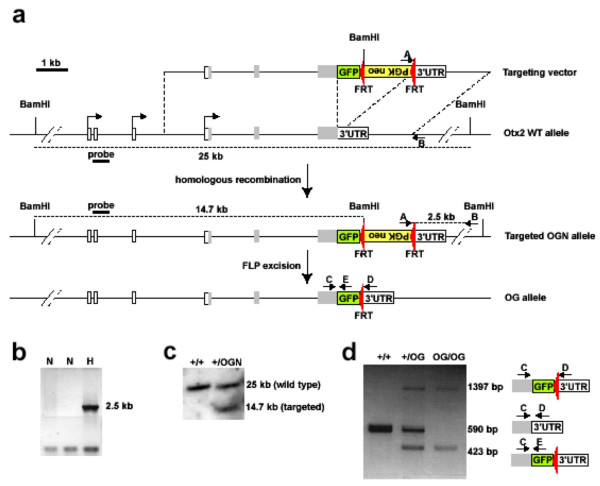
**Generation of the *Otx2*^OG ^allele. a. **The structure of targeting vector (first line), *Otx2 *wild type locus (second line), targeted *OGN *allele after homologous recombination (third line) and *OG *allele after removal of the *Neo *cassette by FLP recombinase (fourth line) is presented. Gray boxes are *Otx2 *coding regions, whites boxes are *Otx2 *5' and 3' UTR regions, yellow box is *PGK-Neo *selection cassette, green box is *muGFP *cDNA. Red triangles are *FRT *sites. Bent arrows symbolize the three main transcription starts sites known for *Otx2 *gene [19, 32]. Dotted lines show BamH I fragments detected by southern blot analysis using probe represented by a thick line. PCR primers used are indicated by arrows. Product obtained with A and B primers is shown. Scale bar and sizes of fragments are indicated. **b. **PCR analysis of Neo^R ^ES clones using primers A and B showing two non-homologous (N) and one homologous (H) recombinants. **c. **Southern blot analysis using BamH I digested genomic DNA and probe indicated in **a **of wild type and homologous recombinant clones. Genotypes are indicated. **d. **PCR genotyping of mice produced from *Otx2*^*+/OG *^ES cells. Analyse was done using C, D and E primers. Sizes, schematic representations of amplified fragments (see part **a **for legend) and deduced genotypes are indicated.

In order to clearly identify Otx2 expressing cells during mouse development and throughout life, we created a new allele bearing the MuGFP coding sequence [21] in frame with Otx2 coding sequence (Figure 1). We had previously shown that fusing the GFP polypeptide both at N- and C-terminus of Otx2 does not modify its *in vitro *localization, DNA binding, and transcription properties [22]. We chose a C-terminal GFP fusion, and put an excisable neo selection cassette immediately downstream the GFP stop codon. Since subtle modifications of the Otx2 3'UTR coding region result in impaired mRNA translation in early embryos [16], we took particular care to keep this part of the gene as intact as possible. After homologous recombination of the targeting molecule in ES cells, neo selection cassette was removed by flp recombinase mediated excision, leaving behind a single FRT site between GFP stop codon and *Otx2 *3'UTR (Figure 1a). All steps of homologous recombination and neo excision were monitored by appropriate PCR and southern blot analyses (Figure 1b–d).

Recombinant ES cells were injected into recipient blastocysts to generate the *Otx2*^*+/Otx2-GFP *^transgenic line. Chimaeric males were obtained who transmitted the *Otx2*^*Otx2-GFP *^allele (thereafter referred to as *Otx2*^*OG *^allele) to their offspring.

### Hypomorphic phenotypes in *Otx2*^*OG/OG *^animals

**Figure 2:**
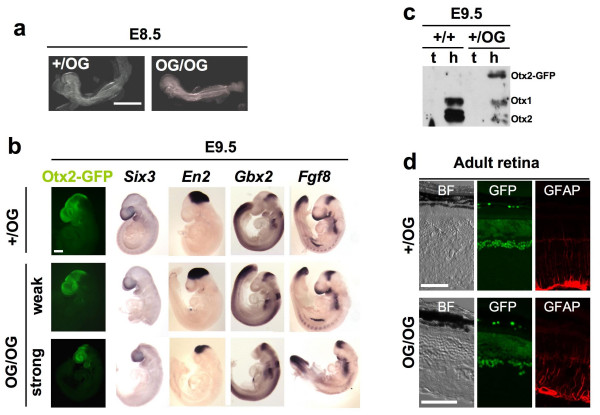
**Characterization of *Otx2*^*OG/OG *^homozygous embryos. a**. Morphology of *Otx2*^*OG/OG *^and control *Otx2*^*+/OG *^animals at E8.5. **b. **Analysis of Otx2-GFP and brain markers in *Otx2*^*OG/OG *^and control *Otx2*^*+/OG *^E9.5 embryos. Otx2-GFP is seen by direct GFP fluorescence visualisation and *Six3*, *En2*, *Gbx2 *and *Fgf8 *mRNAs are detected by whole-mount *in situ *hybridization. For E9.5 *Otx2*^*OG/OG *^animals, upper and lower panels show embryos with weak and strong abnormalities respectively. Anterior is leftward. Scale bar: 500 μm. **c. **Detection of Otx2, Otx2-GFP and Otx1 proteins by western blot analysis of nuclear extracts from tails and heads of *Otx2*^*+/+ *^and *Otx2*^*+/OG *^E9.5 embryos using an anti-Otx antibody. **d. **Normal structure of the retina in surviving *Otx2*^*OG/OG *^adults. Comparison of bright field (BF left panels), GFP fluorescence (middle panels) and GFAP expression (right panels) in adult retinas of the indicated genotype. Scale bars : 50 μm.

Unexpectedly, the breeding of *Otx2*^*+/OG *^mice yielded only 4% living homozygous and fertile *Otx2*^*OG/OG *^offspring (Table 1), meaning the *Otx2*^*OG *^allele is hypomorphic. This was surprising for two reasons. First, Otx2 and Otx2-GFP protein display identical activities *in vitro *[22]. Second, *Otx2*^*OG/+ *^× *Otx2*^*+/+ *^crosses yielded 50% heterozygous animals (Table 1), whereas in the 129/Sv background, only 30% hemizygous *Otx2*^*+/- *^newborn survive [10]. To check for defects in homozygous embryos, mice were sacrificed at various gestational stages. Homozygous embryos were found in mendelian proportions up to birth (see Table 1), but showed variable facial abnormalities (Figure 2b). This evoked a problem of gene dosage or protein activity. Western blot analysis of E9.5 heterozygous embryos showed comparable amount of Otx2 and Otx2-GFP proteins, indicating a similar expression level of both alleles (Figure 2c). Thus, activity rather than quantity of Otx2-GFP protein must be rate limiting, though subtly since it is sufficient to achieve complete mouse development in a small proportion of animals.

**Table 1:** Genotypes of offspring obtained from Otx2^+/OG ^× Otx2^+/+ ^and Otx2^+/OG ^× Otx2^+/OG ^intercrosses at various stages of development.

	**Genotype**	**+/+**	**+/OG**	**OG/OG**	**Total**
**Intercrosses**	**Stage**				
**+/OG × +/+**	Post-natal	149	146		295
	*%*	*50,5*	*49,5*		
**+/OG × +/OG**	E7.5	0	2	5	
	E8.5	11	23	16	
	E9.5	23	56	17	
	E13.5	3	4	3	
	E15.5	1	6	1	
	E17.5	2	5	4	
	E18.5	4	2	1	
	Ante natal	44	98	47	189
	*%*	*23,3*	*51,9*	*24,9*	
	Post-natal (P10–P15)	28	47	3	78
	*%*	*35,9*	*60,3*	*3,8*	

To understand in which manner and when this activity could be limiting, we explored the major early functions of *Otx2 *in homozygous *Otx2*^*OG/OG *^embryos. It is known that this gene controls anterior visceral endoderm (AVE) movement and subsequent gastrulation [18], but morphological examination showed no obvious difference between *Otx2*^*OG/OG *^and *Otx2*^*OG/+ *^embryos up to E8.5 (Figure 2a). This indicates that AVE migration and gastrulation proceeded normally. By contrast, we could observe variable brain reduction from E9.5 on (Figure 2b). *Otx2 *is required in the anterior neuroectoderm to properly receive signals from the anterior neural ridge (ANR) and to maintain forebrain development [23]. *Six3 *is a good indicator of forebrain response to ANR induction. In E9.5 *Otx2*^*OG/OG *^embryos of mild or strong phenotype, *Six3 *was expressed, confirming normal forebrain induction and maintenance (Figure 2b). As *Otx2 *itself monitors forebrain maintenance, Otx2-GFP fluorescence present in every phenotype confirmed an established forebrain identity. It is well known that *Otx2 *controls the compartmentalization and maturation of the forebrain and midbrain [24]. In particular, it is necessary to set the midbrain-hindbrain boundary (MHB). Although a slight anterior shift of Fgf8 expression could not be completely excluded, *Fgf8 *and *Gbx2 *pattern showed no obvious alteration (Figure 2b), indicating that the MHB was correctly established. On the contrary, the *En2 *domain, which normally labels mesencephalon, appeared shifted close to the telencephalon and was reduced in strong phenotypes, showing partial or complete disappearance of diencephalon and mesencephalon respectively – in all cases, however, *Six3 *expression showed that telencephalon developed normally (Figure 2b). Altogether, these data demonstrate that Otx2-GFP protein activity possesses most essential activities of native Otx2, but that it may in some instances be rate limiting for diencephalon and mesencephalon maturation. Interestingly, analysis of surviving homozygous *Otx2*^*OG/OG *^adults showed no obvious difference with heterozygous animals. For instance photoreceptor cells of the retina, which are highly sensitive to *Otx2 *expression [9], appeared normal in *Otx2*^*OG/OG *^adults (Figure 2d). To assay for photoreceptor cells health status, we performed immunostaining of the glial fibrillary acidic protein (GFAP) in glial Müller cells. This protein is up regulated in Müller cells in a variety of degenerative conditions [25]. The absence of marked GFAP induction in *Otx2*^*OG/OG *^retina confirmed that photoreceptors were healthy thus demonstrating the functionality of Otx2-GFP protein in these neurons. For the sake of animal care simplicity, heterozygous animals were used for the continuation of this study.

### Expression pattern in *Otx2*^*+/OG *^animals

**Figure 3:**
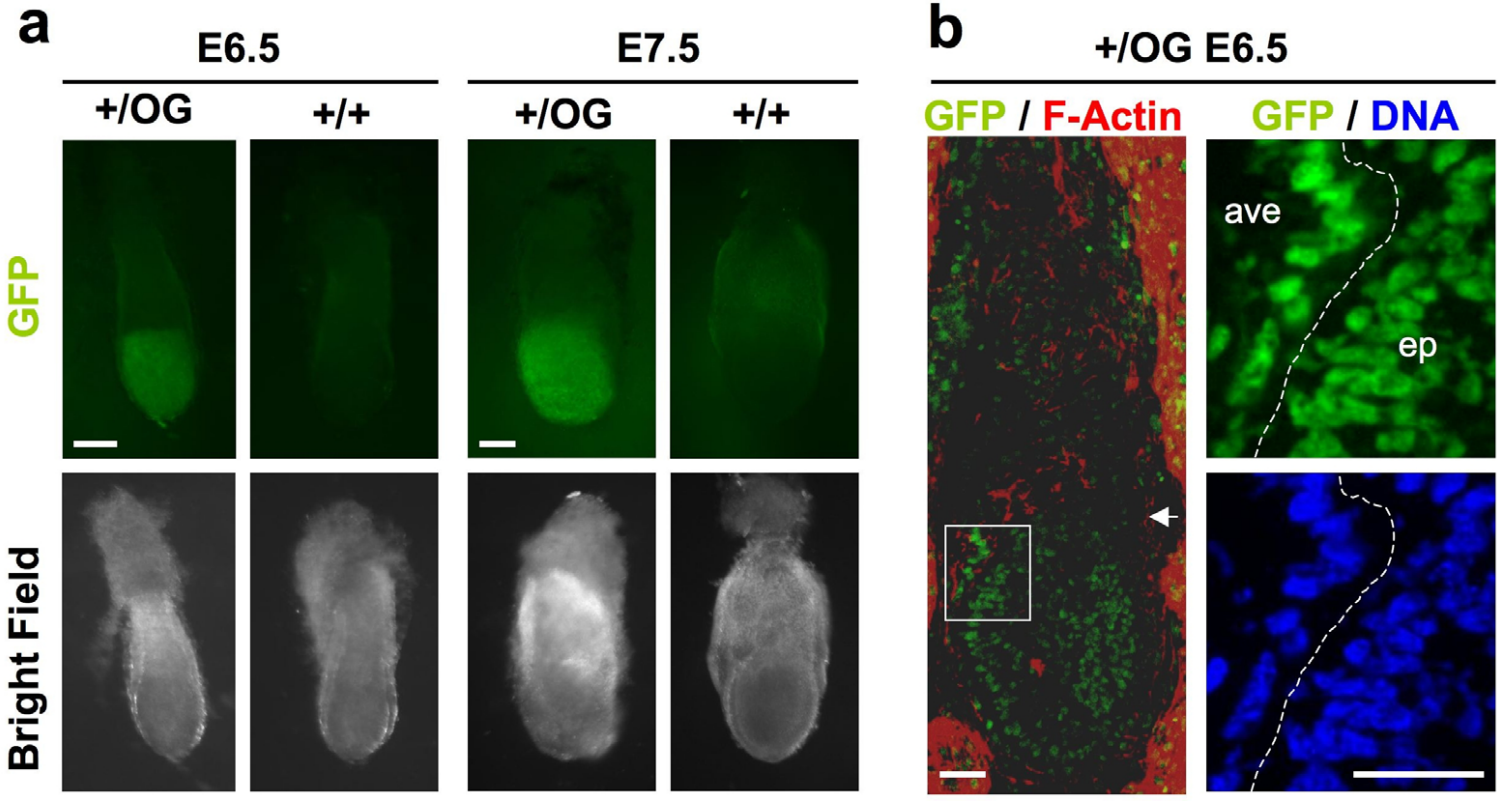
**Early *Otx2-GFP *expression in epiblast and AVE. a. **GFP fluorescence and transmission observations of *Otx2*^*+/OG *^and control *Otx2*^*+/+ *^embryos at E6.5 and E7.5. Anterior is leftward. Scale bars: 100 μm. **b. **Direct visualisation of GFP fluorescence (green) and immunofluorescent staining of F-actin (Phalloidin – red) and DNA (Hoechst – blue) on 10 μm thin uterus sections containing E6.5 *Otx2*^*+/OG *^embryo. Green staining in and around the extraembryonic part of the conceptus is not due to GFP fluorescence but to autofluorescence of decidual and blood cells that are present in the tissue. Images on the right correspond to magnification of white box. Arrow indicates the boundary between embryonic and extra-embryonic part of the conceptus. Dotted line delimits the frontier between anterior visceral endoderm (ave) and epiblast (ep). Anterior is leftward. Scale bars: 50 μm.

To test whether the Otx2^*+/OG *^mouse line is an accurate reporter of *Otx2 *gene activity, we analyzed Otx2-GFP expression pattern. First, early expression of the transgenic allele was studied by direct fluorescence observation of E6.5 and E7.5 embryos. As expected, strong GFP fluorescence was detected in the embryonic part of E6.5 Otx2^*+/OG *^embryos and became regionalized toward the anterior pole in E7.5 embryos (Figure 3a). As Otx2 translation in epiblast has been shown to be very sensitive to locus modification [16], we wondered whether Otx2-GFP was correctly expressed both in epiblast and anterior visceral endoderm (AVE). To verify this, we observed sections of E6.5 embryos. Indeed, nuclear Otx2-GFP was found in both layers (Figure 3b). This expression pattern matches exactly the one described for endogenous *Otx2 *gene [6].

**Figure 4:**
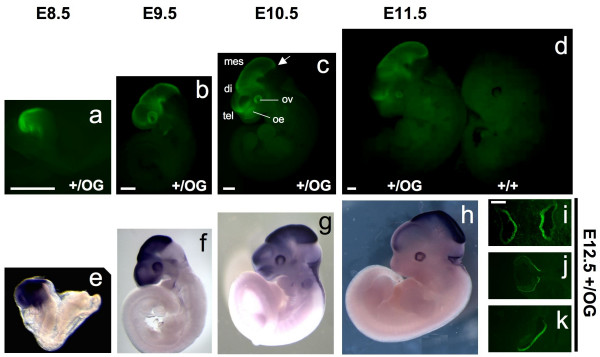
**Dynamics of *Otx2-GFP *expression at mid-gestation**. Direct visualisation of GFP fluorescence of *Otx2*^*+/OG *^embryos and one control *Otx2*^*+/+ *^E11.5 embryo (**a-d**) versus *Otx2 *mRNA localisation detected by whole-mount *in situ *hybridization on *Otx2*^*+/+*^animals (**e-h**) at indicated stages. Anterior is leftward. The arrow indicates the position of the midbrain-hindbrain boundary. Lower right panels: direct detection of GFP fluorescence of transverse sections of E12.5 *Otx2*^*+/OG *^embryo at the level of nasal cavities (**i**), eyecup (**j**) and inner ear (**k**). di, diencephalon; mes, mesencephalon; oe, olfactory epithelium; ov, optic vesicle; tel, telencephalon. Scale bars: 500 μm in **a-d**, 100 μm in **i-l**.

Otx2-GFP protein was then analyzed in developing heterozygous embryos between E8.5 and E12.5 (Figure 4) and compared to *Otx2 *mRNA pattern. Again, the dynamics of Otx2-GFP protein expression paralleled that of *Otx2 *mRNA at all stages. In line with this, we noticed at E10.5 and E11.5 a rapid decrease of Otx2-GFP protein in telencephalic areas where mRNA was down-regulated. Transverse sections across developing sensory organs of E12.5 embryos show GFP signals in olfactory epithelium, eye and inner ear (Figure 4i–k) that are characteristic of Otx2 pattern, indicating that Otx2-GFP products recapitulate endogenous Otx2 expression.

**Figure 5:**
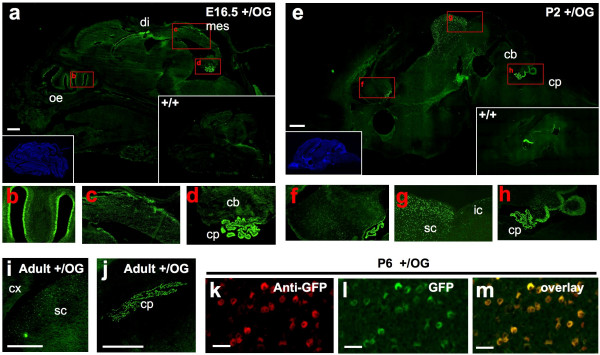
**Efficient *Otx2-GFP *detection during late-development and in adults. a-j. **Direct visualisation of GFP fluorescence on 10μm thin section of E16.5 (**a-d**), P2 (**e-h**) and adult (**i-j**) *Otx2*^*+/OG *^animals. **b-d. **Magnifications of part delimitated by the corresponding red boxes in image **a**. **f-h. **Magnifications of part delimitated by the corresponding red boxes in image **e**. Insets in **a **and **e **are Hoechst staining of the same sections (left) or control fluorescence of 10 μm thin sections of *Otx2*^*+/+ *^animals (right). Anterior is leftward. **k-m. **Comparison of Otx2-GFP detection by  anti-GFP antibody (**k**) or    direct GFP fluorescence  (**l**) in the ventral lateral geniculate nucleus of adult *Otx2*^*+/OG *^animals. cb, cerebellum; cp, choroid plexus; cx, cortex; di, diencephalon; ic, inferior colliculus; mes, mesencephalon; oe, olfactory epithelium; sc, superior colliculus. Scale bars: 500 μm (a, e, i, j) and 10 μm (k-m).

We then addressed Otx2-GFP expression at later stages. This was of particular importance because a previous reporter line failed to express ß-galactosidase beyond E12.5 [6]. In E16.5 sections, as expected again, expression was found in olfactory epithelium, diencephalon, roof of mesencephalon, and choroid plexuses (Figure 5a–d). In P2 brain sections, this expression was maintained. We also identified a diffuse group of Otx2-GFP positive cells in the basal telencephalon (Figure 5f). Furthermore, our line allowed to easily visualize gradients of *Otx2 *expression: such was the case in superior colliculus (SC), where superficial optic layers showed stronger labelling than deep layers, and in cerebellum with high posterior to low anterior labelling of the external granular layer (Figure 5g, h). In adults, very strong labelling could still be seen, among other locations, in choroids plexuses, thalamus as well as SC (Figure 5i–m). Thus, the *Otx2*^OG ^allele appears to be functional from early development till adulthood. To evaluate the sensitivity of detection of the Otx2-GFP protein based on the direct fluorescence emitted by GFP, we compared it with that based on immunostaining using an anti-GFP antibody and a secondary fluorescent antibody. As shown in Figure 5k–m for neurons in the thalamus (ventral lateral geniculate nucleus), both methods detect the same number of Otx2-GFP positive neurons. Similar observations were made in the retina and superior colliculus (not shown). We conclude that virtually all sites of Otx2 protein expression can be recorded by direct observation of the GFP fluorescence in the *Otx2*^*+/OG *^mouse line. Use of amplification reactions might of course further reveal sites of Otx2 expression, particularly at the subcellular level.

### Otx2 is located at the inner face of nuclear envelope in retinal photoreceptors

**Figure 6:**
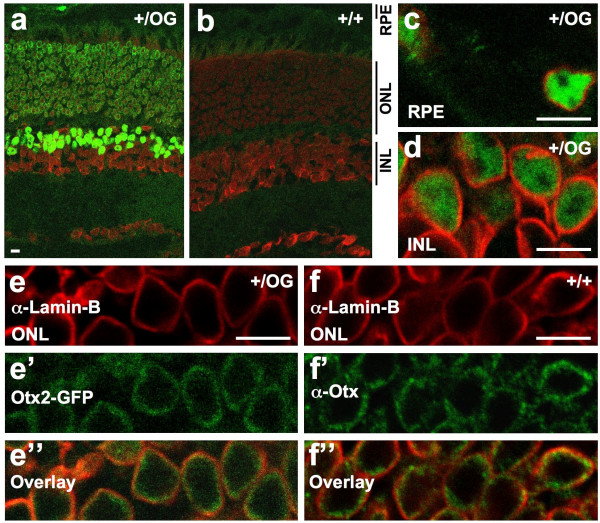
**Regulated Otx2-GFP nuclear localization in adult retina. Confocal microscopy analysis. a, b. **Direct visualisation of GFP fluorescence (green) and propidium iodide staining of DNA (red) on 10 μm thin section of *Otx2*^*+/OG *^(**a**) and control *Otx2*^*+/+ *^(**b**) adult retina. **c-f". **Localisation of Otx2-GFP (direct visualisation of GFP fluorescence – green, **c,d,e',e"**), immunofluorescent staining of nuclear envelope (lamin B – red, **c,d,e,e", f, f"**) and immunofluorescent staining of Otx2 (green – **f', f"**) on 10 μm thin section of *Otx2*^*+/OG *^and wild type adult retina showing retinal pigment epithelium cells (RPE, **c**), outer cells of the inner nuclear layer (INL, **d**) and photoreceptor cells of the outer nuclear layer (ONL, **e-f"**). Image **e" **and **f" **are overlays of images **e **and **e'** and of images **f **and **f' **respectively. Scale bars: 10 μm.

We then wondered whether Otx2-GFP intracellular localization exhibits a regulation similar to the one of native Otx2 protein. In adult mouse retina, a previous study using immunofluorescence with an Otx2 antibody described the protein as nuclear in retinal pigment (RPE) and bipolar cells, but cytoplasmic in photoreceptor (PR) cells [13]. In embryonic chick retina, the protein was found in the nuclei of photoreceptor cells [26]. We therefore used Otx2-GFP to carefully examine cellular localization of Otx2 protein in the retina of adult mice using confocal microscopy. As expected, Otx2-GFP was distributed throughout the nuclear space in RPE cells and in cells located at the outermost part of the internal nuclear layer (INL) (Figure 6a, b), which correspond to bipolar cells [13]. In photoreceptor cells, which have very little cytoplasm around their soma, Otx2-GFP appeared to surround the PR nuclei, but we could not determine whether it was excluded or not from the nucleus. To resolve this issue, we labelled the nuclear envelope with lamin-B and lamin A/C antibodies (Figure 6 and data not shown). This showed diffuse strictly nuclear localization for Otx2-GFP in RPE cells and bipolar cells (Figure 6c, d), but also in PRs – in these cells, the protein concentrated at the periphery of the nuclei, facing the inner nuclear envelope (Figure 6e–e"). To rule out the possibility of an artefact due to GFP as the cause of this unexpected protein distribution in PR cells, we analyzed native Otx2 localization in PRs of wild type mice by immunofluorescence using an anti-Otx antibody (which reacts against both Otx1 and Otx2) together with an anti-lamin-B antibody. As there is no reported *Otx1 *gene expression in PRs, we interpret the anti-Otx signal as the detection of Otx2 protein. We obtained the same result as with Otx2-GFP (Figure 6f–f"), demonstrating that Otx2 protein definitely adopts a perinuclear location in photoreceptors.

## Discussion

We have generated a new mouse line which is unique for direct visualisation of *Otx2 *gene activity. Until now, no other reporter line existed that sustains *Otx2 *driven expression from early embryonic stages to adulthood. As a result of the knock-in fusion strategy in the mouse *Otx2*^*OG/+ *^line, the Otx2-GFP protein is expressed at physiological levels and appears to display most activities and subcellular localization as normal Otx2 protein. In addition, it provides a straightforward mean to discriminate Otx2- from Otx1-expressing cells throughout the mouse brain. Homozygous *Otx2*^*OG/OG *^animals may develop normally but often show mild diencephalic and mesencephalic maturation defects. Interestingly, this reveals that these structures are the most demanding ones for *Otx2 *activity, in line with the frequent ocular and brain defects found in heterozygous mice and human mutants [8,27]. The strong signal to noise ratio of the GFP provides a convenient readout of the *Otx2 *gene dynamics of expression even in living animals. Furthermore, using the *Otx2*^*OG *^allele in combination with modified versions of genes controlling anterior neuroectoderm patterning and development will provide a simple way to monitor normal and perturbed cellular movements.

There is increasing evidence that beyond elaborated control of expression through regulation of transcription by complex enhancers, developmental genes may see their function further modulated by other posttranscriptional mechanisms. Here, the expression of GFP-tagged Otx2 from its endogenous locus helped us to unravel cell-specific control of protein localization within the retina. Using the mouse *Otx2*^*OG/+ *^line, we disclosed that in adult PR cells, Otx2 protein is not cytoplasmic as previously thought, but restricted to a small volume at the inner periphery of nuclei. Interestingly, similar tight regulation of localization was found for the rod photoreceptor specific nuclear receptor Nr2e3 [28]. The significance of such an unusual location for a transcription factor is presently mysterious. The inner face of nuclear envelope gathers silent heterochromatin but also highly expressed DNA domains at the vicinity of nuclear pores [29]. The protein could be sequestrated at the periphery of the PR nucleus in order to regulate the amount of free active Otx2 in the nucleoplasm. Alternatively, all Otx2 proteins at the periphery of the nucleus could be transcriptionally active, bringing loops of DNA containing Otx2-regulated genes to the vicinity of nuclear pores to facilitate mRNA export. Indeed, the chromatin appears to be least condensed at the nuclear periphery of photoreceptor cells [30]. Fish analysis of Otx2 target gene should test this hypothesis. Another possibility is that a specific fraction of chromatin resides at the edge of the nucleus, to which Otx2 and other transcription factors, such as Nr2e3 would be associated. Among the questions raised by this study are the following: when does this pattern take place during PR differentiation and does it have a specific role in PR genetic expression and function ? Conditional ablation of Otx2 function in mature PR will certainly bring interesting answers to these questions.

## Conclusion

The methodology presented here describes the GFP tagging of an important transcription factor from its own gene locus. This leads to the creation of transgenic animals where both tissue and cell specific gene expression as well as precise intracellular protein localization can be easily followed. The established line allows to monitor the complete expression pattern of *Otx2 *gene during mouse life without any interference from *Otx1 *gene products. Its sensitivity leads us to identify peculiar aspects of Otx2 protein expression and cellular localization. This last aspect reveals a new level of regulation for developmental gene products – cell-type dependent control of intra-nuclear distribution – which should be taken into account in future studies.

## Methods

### Generation of the targeting vector, mouse lines production and genotyping

For *Otx2*^*OG *^targeting molecule, a 2.5 kb genomic fragment encompassing the full 3'UTR of *Otx2 *gene and 1.5 kb downstream sequence was amplified using primers 5'-ACGTACTAGTagacctgtagaagctat and 5'-ACGTACTAGTaagtcttgactaggagt and inserted after Spe I-Nhe I restriction into the Spe I site of pOtx2-GFP plasmid [22], immediately downstream the BamHI site at the 3' end of GFP sequence. A BamH I-Bgl II PGK-neo cassette flanked by FRT sites [10] was inserted into the above BamH I site, resulting in pOtx-GFP-neo-3' plasmid. A 3.3 kb EcoR I – Aat II *Otx2 *genomic fragment encompassing *Otx2 *exon 2 and part of exon 3 was then substituted to the 5' *Otx2 *cDNA EcoR I-Aat II fragment of pOtx-GFP-neo-3' plasmid. The 5' homology region was further extended by inserting a neighbouring 2.3 kb EcoRI *Otx2 *genomic fragment encompassing *Otx2 *exon 1C, into the unique EcoR I site of the above plasmid. The Not I linearized targeting plasmid was electroporated into ENS ES cells [31], homologous recombinant clones were identified by PCR using primers A and B and confirmed by southern blot analysis of BamH I digested genomic DNA with the indicated probe (Figure 1c). One positive clone was transfected with a plasmid (pFlp-puro) expressing the flp recombinase to remove the neo cassette. Mice were produced by standard blastocyst injection procedure. Genotypes were assessed by PCR using the primers C, D and E (Figure 1d). Animals were maintained in the 129/Sv background. Day of vaginal plug was taken as E0.5. All animals were handled in accordance with French regulation. Protocols were approved by CREEA, the local ethic committee for animal experimentation.

### Primers

A : 5'- CCTACCCGGTAGAATTGAC

B : 5'- ACTAGGAGTGGCCATCAGA

C : 5'- GCTGGCTCAACTTCCTACT

D : 5'- TTGGTTGCATGTCGCTAGAA

E : 5'- ACCCTCTCCACTGACAGAA

### Analyses of embryos, brains and retinas

For whole mount analyses, samples were dissected in PBS and directly visualised for GFP fluorescence or fixed in 4% paraformaldehyde (PFA) for *in situ *analyses. For sections analyses, samples were dissected in PBS, directly frozen on dry ice for E6.5 or fixed 1 hour in 4% PFA at 4°C, rinsed 3 times in PBS, protected over-night in 30% sucrose and frozen in cryomount. 10 μm section were made, fixed 10 minutes in 4% PFA for E6.5, rinsed 3 times in PBS and mounted in Gel/Mount (Biomeda, CA, USA) or processed before with Hoechst (Ref. 33258, Sigma-Aldrich, Saint Louis, USA) staining (1:200, 5 minutes), Phalloidin (Ref. A34055, Molecular Probes, OR, USA) staining (1:50, 1 hour), Propidium Iodide (Ref. P4170, Sigma-Aldrich, Saint Louis, USA) staining (1:10000, 15 minutes) or Immunostaining followed by 3 times rinsed in PBS. *In situ *hybridization and Immunofluorescence were done as previously described [10]. Probes for *in situ *hybridization are: 0.15 kb *Otx2 *exon2; *Otx2*, *Six3*, *En2*, *Fgf8 *and *Gbx2 *probes were gifts from S.L. Ang, A.P. McMahon, E.J. Robertson, G. Martin and A. Joyner, respectively. Primary antibodies used: 1:100 Goat anti-lamin B (M-20): sc-6217 (Santa Cruz Biotechnology, Santa Cruz, USA), 1:300 Rat polyclonal anti-Otx (gift from M. Wassef), 1:500 Rabbit anti-GFP (Invitrogen, Carlsbad CA, USA) and 1:200 Goat anti-GFAP (Santa Cruz Biotechnology).

### Microscope analysis

Images were done with fluorescent stereomicroscope "Lumar", wide field microscope "Axioplan" or "Axiovert" and confocal microscope "LSM 510" from Zeiss, Jena, Germany.

### Western blot analysis

Extraction of nuclear proteins from E9.5 head (Region in front of MHB) and tail (Region of the most posterior part of the embryo in volume equivalent of the region of the head) was done as previously described [22]. Extracts (15 μg) were subjected to electrophoresis on 6 to 18% gradient gel and transferred to nitrocellulose according to standard protocol. Rat anti-Otx antibody was used at 1:2000 dilution.

## Abbreviations

ANR: anterior neural ridge. AVE: anterior visceral endoderm. CNS: central nervous system. E: Embryonic day. ES: Embryonic Stem Cells. GFP: green fluorescent protein. INL: inner nuclear layer. ISH: *in situ *hybridization. MHB: midbrain-hindbrain boundary. ONL: outer nuclear layer. PR: photoreceptor. RPE: retinal pigment epithelium. SC: superior colliculus.

## Authors' contributions

NF was involved in designing the study, performed microscopy, histology, ISH, western blot analysis and helped to draft the manuscript. CLG and FB raised the line and performed genotyping, statistics and microscopy. PG did the vLGN immunostaining and improved the manuscript. SV performed the homologous recombination of targeting molecule in ES cells and neo cassette excision. GC started to raise the line and performed initial fluorescence analyses. TL conceived and supervised the study, generated the constructs, performed genotyping, statistics and microscopy and drafted the manuscript. All authors have read and approved the final manuscript.
